# Bread and Other Edible Agents of Mental Disease

**DOI:** 10.3389/fnhum.2016.00130

**Published:** 2016-03-29

**Authors:** Paola Bressan, Peter Kramer

**Affiliations:** Department of General Psychology, University of PaduaPadova, Italy

**Keywords:** exorphins, food opioids, celiac disease, gluten sensitivity, gluten-free diet, microbiota, schizophrenia, autism

## Abstract

Perhaps because gastroenterology, immunology, toxicology, and the nutrition and agricultural sciences are outside of their competence and responsibility, psychologists and psychiatrists typically fail to appreciate the impact that food can have on their patients’ condition. Here we attempt to help correct this situation by reviewing, in non-technical, plain English, how cereal grains—the world’s most abundant food source—can affect human behavior and mental health. We present the implications for the psychological sciences of the findings that, in all of us, bread (1) makes the gut more permeable and can thus encourage the migration of food particles to sites where they are not expected, prompting the immune system to attack both these particles and brain-relevant substances that resemble them, and (2) releases opioid-like compounds, capable of causing mental derangement if they make it to the brain. A grain-free diet, although difficult to maintain (especially for those that need it the most), could improve the mental health of many and be a complete cure for others.

## Introduction

“Give us this day our daily bread (…) but deliver us from evil”

*—Matthew 6:11, 13*

About 12,000 years ago, when the last ice age came to an end, the rapid change in climate decimated our traditional food sources, especially large game. Possibly in response to that, in the fertile crescent of the Middle East (roughly the areas comprising the Levant and the Tigris and Euphrates valleys) we began to practice agriculture and animal domestication. Within a few thousand years both had independently started on at least four different continents (Murphy, 2007), stabilizing and increasing our food supply to such an extent that the human population exploded. Yet the agricultural revolution not only increased the availability of food, but also radically changed its nature: cereal grain products, to which we were largely unaccustomed, quickly took center stage. This article illustrates the surprising relevance of this diet change to neuroscientists, psychologists, and psychiatrists.

That the association between humans and grains paid off nicely for both is beyond dispute. Each partner helped the other reproduce, multiply, and ultimately conquer vast patches of the earth. Each partner coevolved with the other, adapting to it. For example, wheat progressively became shorter in response to our own preference for crops easier to harvest and less vulnerable to wind. At the same time, our faces, jaws, and teeth progressively became smaller in response to the soft texture of bread (Larsen, 1995). Thus we domesticated grain, and in return grain domesticated us (Murphy, 2007).

Yet the agricultural revolution may have spelled trouble. Tellingly, whenever diets based on grain replaced the traditional diets of hunter-gatherers, lifespan and stature decreased—while infant mortality, infectious diseases, bone mineral disorders, and the frequency of dental caries increased (Cohen, 1987). Some of these problems were never totally overcome. For example, despite a gradual increase in stature beginning 4,000 years ago, when diets became more varied again, on average we are still about 3 cm shorter than our pre-agriculture ancestors (Murphy, 2007). The coevolution between humans and grain brought on genetic changes in both parties, but did not render grain a more suitable food for us than it originally was.

One of the first hints that these circumstances could have implications for the psychological sciences was the observation that, in several countries, hospitalization rates for schizophrenia during World War II dropped in direct proportion to wheat shortages. In the United States, where over that same period the consumption of wheat rose rather than diminished, such rates increased instead (Dohan, 1966a,b). In South Pacific islands with a traditionally low consumption of wheat, schizophrenia rose dramatically (roughly, from 1 out of 30,000 to 1 out of 100) when Western grain products were introduced (Dohan et al., 1984).

There is now substantial evidence that, depending on genes carried by over one third of us and on seemingly irrelevant factors such as a previous viral infection, eating bread can adversely affect our body and brain. This article reviews the evidence for a wide readership in non-technical, plain English. The next three sections present the implications for the psychological sciences of the facts that bread (1) increases the permeability of the gut and likely of the blood-brain barrier in all of us, (2) sets off an immune reaction in those of us who are genetically predisposed, and (3) breaks down, during digestion, in fragments with opioid activity. The final section discusses whether a change in diet could possibly cure patients with mental illness.

## Grasses, Grains, and Poisons

Grains are the seeds of grasses. Grasses may or may not have evolved to let their seeds be eaten (Janzen, 1984), but certainly not to let them be digested into pieces that will be incapable of passing on the plant’s genes. Grasses cannot defend themselves by fleeing or fighting, they have no thorns, they carry no protective hard shell around their seeds; like most plants, however, they produce toxins. Plants have engineered a wide variety of poisons—over 50,000 defensive compounds have been identified so far (Kennedy and Wightman, 2011)—to deter, harm, or kill the creatures that feed on them. These creatures, in turn, have evolved an arsenal of countermeasures, including mechanisms to detect (for example, bitter taste receptors) and detoxify such poisons as much as possible (Hagen et al., 2009).

Understandably, self-defense proteins are especially concentrated in plants’ most precious fraction—the seeds. Ironically, of the three separate genomes that modern wheat contains from the spontaneous cross-fertilization of three different wild species (e.g., Murphy, 2007), the genome responsible for the best quality bread is associated with the most toxic proteins (Kucek et al., 2015). These are capable, at least in rodents, of crossing both the gut and the blood-brain barriers (Broadwell et al., 1988) and interfere, among other things (Pusztai et al., 1993), with the action of nerve growth factor (Hashimoto and Hagino, 1989). In pasta, many of these proteins—though highly resistant to digestion—are lost in the salted water during cooking, hence they do not make it to the final dish (Mamone et al., 2015). Yet they can still be found in beer and pre-steamed couscous (Flodrová et al., 2015) and can be inhaled from raw flour (Walusiak et al., 2004).

Seeds are also equipped with proteins designed to provide ready-made nourishment for the future seedling. The kit of storage proteins in barley, rye, and particularly wheat, known as gluten (“glue” in Latin), has turned out to have special value for us. As bread dough is kneaded, gluten forms an elastic network that traps the gases produced by yeast during fermentation; this allows the dough to rise and to expand during baking. The spectacular success of wheat relative to barley and rye has mainly hinged on the ease with which a light, porous, optimally chewy bread can be obtained from its flour.

Unfortunately, gluten has proven to be toxic for a proportion of people that in the last few decades has been constantly rising (Rubio-Tapia et al., 2009). Indeed, the wheat varieties that contain the most detrimental type of gluten have become more common (van den Broeck et al., 2010). This is particularly worrying given that gluten is not only naturally present in bread, cake, pasta, pizza, and beer, but it is—for its binding and thickening properties—also added to an impressive variety of other products. A survey of Australian supermarkets found gluten in nearly 2,000 different food items, ranging from sauces to processed meats, and over 100 non-foods, from pain relievers to shampoos (Atchison et al., 2010). Yet gluten triggers some action as soon as it turns up in the gut—not only in a few sensitive people, but in all of us.

## Holes in Our Gut

A post-mortem study of 82 schizophrenia patients found rates of stomach, small intestine, and large intestine inflammation as impressive as respectively 50%, 88%, and 92% (Buscaino, 1953; cited in Buscaino, 1978). The association between gastrointestinal pathologies and psychiatric disorders had already been noticed at least 2,000 years ago and has been confirmed repeatedly (for a brief review see Severance et al., 2015).

An unhealthy gut may open up our body to harmful bacteria, toxins, and undigested pieces of food. In each of us, an intestinal wall whose surface could pave an entire studio apartment (Helander and Fändriks, 2014) faces the challenge of preventing this from happening, while letting water and nutrients through. This feat is accomplished via a sophisticated barrier, where the opening and closing of the junctions between the wall’s cells are adjusted flexibly (Bischoff et al., 2014). Other than that, this architecture might serve as an emergency line of defense against pathogenic microbes (Fasano et al., 1997). The part of the gut that immediately follows the stomach, the small intestine, is in fact kept virtually sterile—bacteria are removed by the peristaltic movements of the gut before they can multiply (Dixon, 1960). Any abnormal presence of microbes triggers the release of the protein *zonulin*, which widens the junctions between cells so that water can seep into the intestine and flush out bacteria via loose bowel movements (El Asmar et al., 2002).

Producing diarrhea is just one exceptional job among the many, less conspicuous daily ones that zonulin is believed to perform. Importantly, regulation of intestinal permeability grants or vetoes the passage of large molecules and immune cells. For still obscure reasons, however, sometimes this mechanism allows partially undigested food components to escape from the gut and reach (a) the inner layer of the intestinal wall, which hosts a large part of the immune system, and (b) the bloodstream. These substances are not expected there and can set in motion a misdirected immune reaction (for a readable account of the details, see Fasano, 2009). Protracted abnormal gut permeability is indeed associated with a wide range of immune-related diseases, and in some animal studies has been shown to precede them, suggesting causation (e.g., Meddings et al., 1999). These diseases include arthritis, asthma, type 1 diabetes, and multiple sclerosis (Fasano, 2012).

It is worth noting that psychological stress worsens gut permeability. Speaking in public does it, with transient effects (Vanuytsel et al., 2014), and early maternal deprivation does it too, with long-term ones (demonstrated in rats: Barreau et al., 2004). Interestingly, psychological stress also worsens gut inflammation (for a brief review, see Daulatzai, 2015), exacerbates immune-related diseases (Dhabhar, 2009), and predicts the onset and severity of mental disorders (Kendler et al., 1999; Carr et al., 2013). Some common spices (Jensen-Jarolim et al., 1998) and food components (e.g., Bischoff et al., 2014) modulate gut permeability too, either increasing it (like fructose, widely used to sweeten commercial beverages) or decreasing it (like the flavonoid quercetin, found in onions and tea). Probably because it is mistaken for a microbial molecule (Fasano et al., 2015), gluten stimulates zonulin release and hence features prominently in the former group (Hollon et al., 2015). Ingestion of an inhibitor of zonulin prevents gluten from raising gut permeability, and a gluten-free diet reduces both zonulin levels and gut permeability (Fasano, 2011). In all of us, zonulin increases the permeability not only of the intestinal wall, but also of other no less interesting barriers—notably the blood-brain one. A toxin mimicking zonulin is actually being studied for its ability to enhance delivery to the brain of drugs such as anticancer agents (Karyekar et al., 2003).

## Immune System Mistakes

After increasing gut permeability and with its help, gluten can make trouble if it happens to cross the outer layer of the intestinal wall and become the target of immune surveillance. The next two subsections explore the consequences of this encounter on our body and on our brain.

### The Many Forms of Wheat Sensitivity

Some people are overtly allergic to wheat (from here on, “wheat” will cover all gluten-containing grains). Minutes to hours after exposure, these individuals develop symptoms such as rashes, headaches, diarrhea, or shortness of breath—a well-known example is baker’s asthma. This *wheat allergy* (Inomata, 2009) engages the part of our immune system that responds quickly against parasitic worms, fungi, and microorganisms. In some of us, however, gluten triggers immune-mediated reactions whose symptoms develop gradually, weeks to years after its introduction in the diet.

In about 1 person out of 100 this hypersensitivity is expressed as *celiac disease*, defined as a chronic immune reaction against one’s own small intestine. Over time, this reaction flattens the intestinal wall (which is normally covered with millions of finger-like protrusions), reducing its surface and thus its ability to absorb important nutrients for both body and brain. If gluten is not withdrawn during childhood, the growth of some cranial bones is altered as well. As a result, over 80% of adult celiacs have unusual facial proportions (Zanchi et al., 2013). Quite typical is an especially high forehead relative either to the middle third of the face or to the forehead of healthy people (see, respectively, Finizio et al., 2005; and Zanchi et al., 2013).

Most people with celiac disease do not know they have it. In a sample of over 5,000 Italian students, for example, the ratio of diagnosed to undiagnosed cases was 1 to 6 (Catassi et al., 1995). In the elderly, celiac disease often goes unrecognized as well, with a mean delay of 17 years from the onset of symptoms to diagnosis (Gasbarrini et al., 2001). Alarmingly, blood markers of the disease have quadrupled in the United States in the last 50 years (Rubio-Tapia et al., 2009) and doubled in Finland in the last 20 (Lohi et al., 2007). Measurements were taken all at once on blood samples collected and frozen decades apart, hence the recent surge in the disease cannot be due to better detection or more lenient diagnostic criteria. Markers also increase within the same group of individuals over time, showing that an abnormal immune response to gluten can suddenly emerge in adulthood (Catassi et al., 2010).

Some people do better on a gluten-free diet and worsen upon a gluten challenge (even under double-blind, randomized, placebo-controlled conditions: Biesiekierski et al., 2011) although they do not meet the criteria of either wheat allergy or celiac disease. This *non-celiac gluten sensitivity* is diagnosed by exclusion, because there are currently no laboratory tests for it. The gut permeability of these people is normal, unlike that of celiacs—but gluten makes it soar just as much as that of celiacs (Hollon et al., 2015). Symptoms emerge hours to days after gluten exposure and are largely extraintestinal; they include headache and eczema but also fatigue and “foggy mind” (Sapone et al., 2012). Other individuals report being sensitive to gluten but actually experience bloating and abdominal pain from wheat’s carbohydrates (Biesiekierski et al., 2013). Many studies on non-celiac gluten sensitivity have not controlled for the presence of these carbohydrates; they can also be found in various vegetables, however, and whether their effects can go beyond mere intestinal discomfort is debatable (for opposing views, see Fasano et al., 2015; De Giorgio et al., 2016).

Over 95% of celiacs carry a specific variant of a gene that is corresponsible for the regulation of the immune system, and about 5% carry another (Diosdado et al., 2005). Crucially, both genes are implicated in the ability of the immune system to distinguish self from non-self. These genes are also present in 30–40% of the general population, however, and of course not all of them develop celiac disease; even monozygotic twins on the same diet can be discordant for it (Greco et al., 2002). Other factors must thus be involved—possibly, simple environmental triggers. These have been shown to range from delivering a baby (Malnick et al., 1998) to contracting a virus or a parasite. In one study, for example, nearly 90% of celiacs, vs. 17% of controls, showed evidence of previous infection with adenovirus (Kagnoff et al., 1987). Because a protein coded by this virus is structurally similar to gluten, it is plausible that in genetically predisposed individuals the initial reaction to the virus may extend to gluten and then to some proteins in our own intestine that resemble both—a process called molecular mimicry (see Kasarda, 1997).

### Wheat and the Mind

Unfortunately, gluten resembles some brain-relevant substances too. *In vitro*, antibodies against gluten removed from human blood attack cerebellar proteins and components of the myelin sheath that insulates nerves (Vojdani et al., 2014). They also attack an enzyme involved in the production of GABA—our prime inhibitory neurotransmitter, whose dysregulation is implicated in both anxiety and depression. In the blood of blood donors, antibodies against wheat or milk and antibodies against these brain-relevant substances have been found to be simultaneously elevated, consistent with the presence of a cross-reaction (Vojdani et al., 2014). Most of us escape it only because our gut and blood-brain barriers are intact—and only as long as they remain so. Antibodies against the brain, triggered by gluten, can cause severe neurological dysfunctions whether or not one is celiac (Hadjivassiliou et al., 2010). Similar antibodies have also been found in the blood of a subgroup of schizophrenia patients; some of them carried blood markers of celiac disease, but others did not (Cascella et al., 2013).

If wheat can affect the brain, it should come as no surprise that it can affect mental health too (for a review, see Jackson et al., 2012a). Exceptionally large epidemiological studies, each involving many thousands of patients, have found that celiac disease is associated with an increased risk of depression (Ludvigsson et al., 2007b) and psychosis (Ludvigsson et al., 2007a). Among individuals with a normal intestinal wall, those who carry blood markers of celiac disease are three times more likely to develop autism in the future, and five times more likely to have already been diagnosed with it (Ludvigsson et al., 2013).

Antibodies against gluten have been found much more often in schizophrenia and autism patients than in the general population or in controls, a result that has been replicated repeatedly (Jackson et al., 2012a). Some figures are stunning, such as a reported presence of antibodies against gluten in 87% of unmedicated autistic children vs. 1% of normal children (Cade et al., 2000).

### Microbial Accomplices

The main gene that predisposes to celiac disease also changes the composition of the microbes in the gut; a notable finding, because we now know that these microbes (collectively known as gut microbiota) are directly capable of shaping our behavior (Dinan et al., 2015; Kramer and Bressan, 2015). Carriers and non-carriers of the gene produce stools with significant bacterial differences at 1 month of age already (Olivares et al., 2015). Among other things, carriers host more clostridia; clostridia tend to be overrepresented in the guts of children with autism (Louis, 2012), and it is suggestive to associate these findings to the epidemiological evidence, discussed earlier, of a larger risk of autism in celiacs.

Gut microbes even appear to play a part in when (and possibly whether!) carriers do develop celiac disease. Because the maturation of our immune system is co-driven by our microbial community (Kranich et al., 2011), it is crucial that the latter develops normally—which could be jeopardized by feeding babies inappropriate foods at an inappropriate time. The microbiota matures enormously in the first 12 months of life, hence it might be important to avoid gluten during this period (Fasano, 2009). Indeed, a double-blind study on young carriers of the celiac gene compared the relevance of early (6 months of age) vs. late (12 months) introduction of gluten in their diets. Early introduction promptly caused loss of tolerance to gluten and set off the development of autoimmunity, arguably via a change in the composition of the still immature microbiota (Sellitto et al., 2012). Indeed, whether or not transgenic mice with the celiac gene in them express the disease has recently been shown to be entirely determined by their guts. Eating gluten started celiac disease in the mice who had been raised without gut microbes, or whose microbiota included pathogens or had been perturbed by antibiotics right after birth—but not in the mice whose microbiota was healthy (Galipeau et al., 2015).

Changes in gut microbiota due to a sudden, massive exposure to wheat products have also been hypothesized to mediate the well-known relationship between immigrant status and schizophrenia (Severance et al., 2014). This might be, for example, the case of people moving to Europe from sub-Saharan Africa, where staple grains do not include wheat and are traditionally broken down via fermentation before being eaten. It is thus entirely possible that bread can be harmful to our mental health not only directly, via some of the proteins it contains; but also indirectly, via its effects on our gut microbes. The causal relationship between eating bread and harboring certain microbes could actually go both ways, as suggested by recent evidence that our craving for certain foods may be driven by the intestinal bacteria that feed on them. Bread is ultimately broken down to sugar, and plenty of microbes thrive on sugar. When not enough of it comes their way, microbes might be able to manipulate their host by inducing bad mood and other painful sensations—relieved only by eating the right stuff (Alcock et al., 2014).

## Bread and Other Drugs

During digestion, gluten is broken down into hundreds to thousands of fragments that are not further dissolved. Some of them resemble morphine extremely much and have thus been named *exorphins* (where “exo” refers to their external origin; Zioudrou et al., 1979). Exorphins are released from other proteins as well—prominently casein, found in milk and very similar to gluten, but also albumin in rice and zein in corn (Teschemacher, 2003). How exorphins affect our behavior, and what happens if they are absorbed from the gut and show up in the brain, are the topics of the next two subsections.

### Exorphins Posing as Endorphins

Like morphine, exorphins bind to opioid receptors that are widely distributed throughout the body—in places as different as the gut, the lungs, the genitals, the various districts of the nervous system. Such receptors are of course meant for our own opioids, endorphins (of internal origin, “endo”). Our body may produce endorphins to reduce pain when we need to continue functioning in spite of injury or stress, as during labor or combat. The “runner’s high”, the state of euphoria experienced by long-distance runners, might capitalize on this mechanism (Boecker et al., 2008; but see also Fuss et al., 2015).

It has been intriguingly suggested that one major function of endorphins would be to protect the organism against starvation in times of stressful, prolonged food scarcity (Margules, 1979, 1988). We know that the same opioid can exert opposite effects depending on which receptor it binds to (Teschemacher, 2003); the key might be whether the receptor happens to sit in the body or in the brain (Margules, 1988). Bound to opioid receptors in the gut, in fact, morphine-like endorphins tend to conserve bodily resources (by inducing constipation and water retention), reduce motor activity, decrease pain, suppress both reproduction hormones and sexual desire. Bound to opioid receptors in the brain, on the contrary, they promote energy expenditure, boosting reactivity and (hyper)activity. The former could be interpreted as a passive, hibernation-like response to seasonal food shortage; the latter as an active, migration-inducing one (Margules, 1988; see also Guisinger, 2003). The potential connection between a malfunctioning opioid system and eating disorders such as anorexia has not gone unnoticed—and it is supported by several lines of evidence (see Yeomans and Gray, 2002). Notably, endorphins are produced on demand, but exorphins are generated at virtually every (modern) meal.

Food exorphins appear to perform their job largely or entirely from the gut. Thus, they should support energy sparing in all kinds of manners (see evidence for some of these in Teschemacher, 2003). Yet exorphins directly bind to the opioid receptors of the brain as well, if they can get there (Kostyra et al., 2004). The question is whether they pass through the intestinal and blood-brain barriers in meaningful amounts. Some authors argue that, if these barriers are healthy, they probably do not (Miner-Williams et al., 2014). This is hardly reassuring, though, given how easily the function of even healthy barriers can be disrupted—be it by stress (Söderholm and Perdue, 2001), dietary components (Ulluwishewa et al., 2011), alcohol (Purohit et al., 2008), or familiar over-the-counter drugs (e.g., Smale and Bjarnason, 2003). Indeed, radioactively labeled gluten proteins fed to rats by stomach tube are later found in the animals’ brains in the form of exorphins (Hemmings, 1978; for related evidence regarding dairy proteins, see Sun and Cade, 1999).

The manufacture of exorphins is incredibly efficient. The nutritionally insignificant intake of 1 g of casein (about two tablespoons of cow milk), for example, produces opioids in large enough amounts to exert physiological effects (Meisel and FitzGerald, 2000). This is remarkable in view of the facts that (a) the opioids from gluten are stronger than those from casein (Zioudrou et al., 1979), and (b) the daily average consumption of gluten in Europe is 10–20 g, with many people exceeding 50 g (Sapone et al., 2012). In the brain of rats, the opioids from casein have been shown to be 10 times more potent than morphine (Herrera-Marschitz et al., 1989). If all exorphins released in the gut made it to the brain, it is hard to see how we could keep functioning.

Opioids are involved in both the palatability and rewarding aspects of food, hence they play a major role in food cravings and food addiction (for a review, see Yeomans and Gray, 2002). The opioid antagonist naloxone drastically reduces the intake of preferred foods, but not of nonpreferred ones, in rats (Glass et al., 1996; see also Boggiano et al., 2005). Naltrexone, which is much like naloxone but it lasts longer and can be taken by mouth, suppresses binge eating in humans (Marrazzi et al., 1995). Indeed, people who have first ingested naltrexone (against placebo: Yeomans and Gray, 1997) rate a bowl of pasta as less pleasant, and eat less of it. Tellingly, naloxone is famed for its ability to counteract the effects of an overdose of heroin, a potent derivative of morphine, and naltrexone is used in the treatment of heroin dependence.

Foods that contain exorphins, such as wheat and dairy products, have indeed a reputation for being rewarding and people find it extremely hard to give them up. The addictive properties of milk were arguably designed by evolution to gratify suckling young. The gut of newborns is highly permeable—not only to the mother’s antibodies as an aid to their still immature immune system, but also to milk opioids (see Teschemacher, 2003). Yet, production of the enzyme for properly digesting milk is genetically programmed to stop after weaning. Regular intake of milk by adults is evolutionarily novel and only started with animal domestication; it was permitted by a mutation of this enzyme in populations that kept cattle. Interestingly and perhaps worryingly, the opioids in bovine milk are 10 times stronger than those in human milk (Herrera-Marschitz et al., 1989). This might not be extraneous to the fact that about half of children up to 4 years of age need their milk bottle to fall asleep at night (in Thailand: Sawasdivorn et al., 2008). Note that, as mentioned, the opioids in wheat are even stronger than those in bovine milk (Zioudrou et al., 1979).

Arguably, foodstuffs whose digestion releases exorphins are preferred exactly because of their drug-like properties. It has been speculated, in fact, that this chemical reward might have been one incentive for the initial adoption of agriculture (Wadley and Martin, 1993). Why cereals rapidly and extensively replaced traditional foods even though they were less nutritious and required more labor has been widely regarded as a puzzle. Also, cultivation of cereals continued even when the abundance of more easily processed foodstuffs—such as meat, tubers, and fruit—rendered it unnecessary (see Murphy, 2007). A clue could be the fact that all major civilizations, in every inhabited continent, arose in groups that practiced cereal agriculture and not in groups that only cultivated tubers and vegetables or had no agriculture at all. According to Wadley and Martin’s rather audacious hypothesis, daily opioid self-administration could have increased people’s tolerance of crowded sedentary conditions, of regular work, of subjugation by rulers. If so, cereals might have ultimately helped the development of civilization.

### Too Much Exorphin in the Wrong Place

Not all individuals handle these substances the same way. For example, abnormally high levels of milk and/or wheat exorphins have been found in the urine (Hole et al., 1979) and blood (Drysdale et al., 1982) of schizophrenia patients and in the urine (e.g., Sokolov et al., 2014; but see Cass et al., 2008) of autistic children. When purified and injected in the brain of rats, these substances made the rats behave in strikingly odd ways—very restless at first and then inactive and hyperdefensive. Among other things, the rats paid no attention to a ringing bell, in suggestive similarity to the apparent deafness often observed in children with autism (Sun and Cade, 1999; Cade et al., 2000). Interestingly for the nonpatients among us, exorphins coming from healthy people’s blood had on rats effects that were weaker and briefer but otherwise similar (Drysdale et al., 1982).

Besides producing behavioral disorders similar to those seen in schizophrenia and autism (such as decreased social interaction, reduced pain sensitivity, uncontrolled motor activity: Sun and Cade, 1999), exorphins activate in rats the same brain regions that are affected in schizophrenia and autism. The disruptive effects they exert on visual and auditory areas are consistent with typical malfunctionings such as hallucinations in schizophrenia (Sun et al., 1999). So perhaps it is no coincidence that a recent case report of an adult patient has described complete resolution of highly unsettling visual and auditory hallucinations, experienced daily from early childhood, upon removal of gluten from the diet (Genuis and Lobo, 2014).

The effects of food exorphins on behavior (for a comprehensive review, see Lister et al., 2015) and on the brain (Sun et al., 1999) are reversed by treating the rats with opioid antagonists. Naloxone has also been shown to temporarily erase psychotic symptoms, especially hallucinations, in schizophrenia patients (Emrich et al., 1977; Jørgensen and Cappelen, 1982). Naltrexone benefits some children with autism (Roy et al., 2015), arguably by blocking a brain opioid activity that might be abnormally high in these children (Sahley and Panksepp, 1987). Attempts to eliminate excess exorphins from the blood of schizophrenia patients via weekly dialysis for 1 year have led to remarkable results as well, with 40% of patients vastly improving or fully recovering from schizophrenia. In some patients who did not get better, the continuous production and absorption of exorphins on a regular diet might have been so large that dialysis failed to reduce their concentration in the blood. Indeed, of the five patients that combined dialysis with a diet devoid of gluten and casein, all either improved significantly or became entirely normal (Cade et al., 2000).

In psychotic children (Gillberg et al., 1985), schizophrenia patients (Lindström et al., 1986), and women with postpartum psychosis (Lindström et al., 1984), larger-than-normal amounts of exorphins have been detected in the cerebrospinal fluid. Exorphins clearly do not belong there. In the presence of faulty barriers, though, they could migrate from the gut to the blood (prompting an immune reaction) and from there to the cerebrospinal fluid. In people with schizophrenia (unlike in healthy individuals) the more antibodies against gluten one finds in the blood, the more one finds in the cerebrospinal fluid (Severance et al., 2015). This correlation suggests a larger diffusion of antibodies from one place to the other in patients than in nonpatients, pointing to some barrier dysregulation—possibly subtle or transient. It is worth recalling that gluten comes prepackaged with the ability to cause such dysregulation itself.

## Diet as a Cure

Evidence that a diet devoid of wheat (and possibly of dairy as well, given the similarity between gluten and casein) can cure some patients with mental illness has been available for nearly 50 years. Yet because other patients—especially in newer and better studies—have not changed on the diet, such evidence has been variously downplayed, discredited, or dismissed. As a result, the message has made it neither to patients and their caretakers nor to psychologists and psychiatrists. After looking at all of these dietary intervention studies, we have come to believe that this lack of communication is a mistake.

Most studies have been run on schizophrenia patients kept in psychiatric wards, where meals could be tightly supervisioned. Patients on a grain-and-milk-free diet were either discharged or transferred from a locked to an open ward sooner than patients on a grain-rich diet (Dohan et al., 1969; Dohan and Grasberger, 1973). The effect was canceled when, blind to both patients and staff, the grain-and-milk-free diet was supplemented with gluten. A double-blind, placebo-controlled, longitudinal study with very similar results was impressive enough to get into the journal *Science* (Singh and Kay, 1976). Here schizophrenia patients kept on a grain-and-milk-free diet worsened on 30 out of 39 behavioral measures when a “special drink” they were given daily contained gluten and recovered when it contained soy flour instead.

Abstention from gluten and casein, especially when protracted for several months, also benefits a proportion of children with autism spectrum disorders (for a review, see Whiteley et al., 2013). In one study that followed 70 such children who had not previously responded to any therapy, this proportion reached, after 3 months on the diet, an impressive 80% (Cade et al., 2000).

In gluten-sensitive people, long-term wheat consumption could potentially lead to permanent damage (Kalaydjian et al., 2006; Hadjivassiliou et al., 2010); thus one does not necessarily anticipate change in chronic patients. Still, on gluten-free diets, clear improvements in psychiatric symptoms have been observed in severely disturbed schizophrenia patients who were unresponsive to any form of treatment and had spent much of their lives in institutions (Rice et al., 1978; Vlissides et al., 1986; see also Cade et al., 2000). Some of these patients worsened dramatically as soon as gluten was reintroduced.

Improvement in mental health on a gluten-free diet should, of course, only be expected for individuals that have an adverse physical reaction to wheat, expressed for example as gluten-related antibodies. Indeed, in a small study on eight chronic schizophrenia patients who were shown *not* to react to gluten, none improved on a gluten-and-milk-free diet (Potkin et al., 1981). To date, only case studies have focused specifically on the subset of patients that is demonstrably sensitive to wheat. In each and every case, the results of a gluten-free diet have been impressive. Clear improvement has been observed in two patients with schizophrenia (Jackson et al., 2012b) and two with dementia (Lurie et al., 2008). Full recoveries have been separately reported for three patients with severe psychotic symptoms (De Santis et al., 1997; Eaton et al., 2015; Lionetti et al., 2015).

Ironically, the greater the potential benefit of a change in diet, the greater the resistance to it may be (Wadley and Martin, 1993). Grain’s exorphins can create addiction. It has been estimated that half of the people that are hypersensitive crave the very food that causes them harm and experience withdrawal symptoms when they remove it from their diet (Brostoff and Gamlin, 1989). Remarkably, one untreatable, high-security-ward schizophrenia patient who had made a miraculous recovery on a gluten-free diet became violent and extremely disturbed when gluten was reintroduced. At that point, he was unable or unwilling to resume the gluten-free diet that would save him (Vlissides et al., 1986).

## Conclusion

We have shown that in all of us bread makes the gut wall more permeable, encouraging the migration of toxins and undigested food particles to sites where they can alert the immune system. We have shown that in all of us the digestion of grain and dairy generates opioid-like compounds, and that these cause mental derangement if they make it to the brain.

Together, these pieces of evidence prompt the question of why not all of us develop psychotic symptoms on a diet of bread and milk. One plausible answer (Severance et al., 2015) is that the individuals who ultimately come down with these symptoms might be carrying an “immune defect” such that exorphins, once in the blood, either attract too much attention from the immune system or escape detection altogether. The resulting antibodies (in the former case) or the exorphins themselves (in the latter) could then gain access to the brain. They would arrive there from the bloodstream, either directly or via the cerebrospinal fluid, by way of faulty barriers. An alternative idea is that the genetic defect is not in the immune system, but in the enzymes involved in breaking down exorphins in either the gut or the blood (Dohan, 1980; see also Reichelt et al., 1996).

Even though wheat causes them serious harm, most individuals with celiac disease are unaware they have the illness, and for the rest of us no test is currently available that can conclusively reveal whether we are hypersensitive to wheat. The evidence is overpowering, though, that such hypersensitivity can bring with it mental disturbances like schizophrenia, bipolar disorder, depression, anxiety, and autism. Patients who have been demonstrated to be hypersensitive have either improved substantially or even been cured completely of their mental symptoms on a wheat-and-dairy-free diet. For all the others, wheat-sensitivity tests notwithstanding, we advocate trying the elimination of wheat and dairy or at least gluten and casein—for diagnostic purposes if nothing else. Unlike with pharmacological treatments, there have been no reports of harmful side effects. Actually, contrary to common wisdom, whole grains rank next to last in nutrient density among food groups (Cordain et al., 2005); they are nutrient-dense only in their raw, inedible state (Lalonde, 2012). Hence, replacing grains with vegetables, fruit and nuts, meats, and seafood actually increases the vitamin and mineral content of the diet.

Bread is the very symbol of food, and learning that it can threaten our mental wellbeing may come as a shock to many. Yet bread is not alone; like it, other foodstuffs, such as milk, rice, and corn, release exorphins during digestion. Wheat, rice, and corn are the staples of over 4 billion people. Another popular substance, sugar, is prominent in many of our supermarket products and hidden in a myriad others. While not a source of exorphins, sugar provokes the release of endorphins and can induce—with addiction-associated neurophysiological changes—impressive craving, bingeing, and withdrawal problems (Ahmed et al., 2013).

Psychologists and psychiatrists typically pay much attention to their patients’ aberrant eating habits. They may wish to keep an eye on their normal eating habits as well.

## Author Contributions

PK wrote part of the first draft; PB wrote part of the first draft and the final version. Both authors conceived the work, searched and studied the literature, and contributed to the viewpoint that the article expresses.

## Conflict of Interest Statement

The authors declare that the research was conducted in the absence of any commercial or financial relationships that could be construed as a potential conflict of interest. Despite currently receiving royalties from a publication based on the use of dietary intervention for autism and related conditions and being a shareholder of an online company that provides dietary information about the use of a gluten and casein free diet, the Reviewer PW and handling Editor declare that the process nevertheless met the standards of a fair and objective review.
